# Sustainable Polyphenol-Rich Extracts from Agricultural By-Products: Infectivity Inhibition Potential for Human Coronavirus 229E

**DOI:** 10.3390/molecules30081806

**Published:** 2025-04-17

**Authors:** Joan Teichenné, Yaiza Tobajas, Kevin Leonard, Job Tchoumtchoua, Xavier Escoté

**Affiliations:** 1 Eurecat-Centre Tecnològic de Catalunya, Unitat de Nutrició i Salut, 43204 Reus, Spain; yaiza.tobajas@eurecat.org (Y.T.); xavier.escote@urv.cat (X.E.); 2 Biomass Valorisation Platform, Extraction Department, CELABOR Srl, 4650 Herve, Belgium; kevin.leonard@celabor.be (K.L.); job.tchoumtchoua@celabor.be (J.T.); 3 Nutrition and Metabolic Health Research Group, Department of Biochemistry and Biotechnology, Rovira i Virgili University (URV), 43201 Reus, Spain; 4 Institute of Health Pere Virgili (IISPV), 43204 Reus, Spain; 5 Center of Environmental, Food and Toxicological Technology—TecnATox, Rovira i Virgili University, 43201 Reus, Spain; 6 CIBER in Physiopathology of Obesity and Nutrition (CIBEROBN), Carlos III Health Institute, 28029 Madrid, Spain

**Keywords:** viral inhibition, coronavirus, HuCoV-229E, antiviral, polyphenols, agricultural by-products, bioactive compounds, green extraction

## Abstract

Polyphenol-rich extracts derived from agricultural by-products exhibit promising antiviral properties. This study evaluated the antiviral potential of extracts from red onion peels, vineyard prunings, olive prunings and chicory leaves against human coronavirus HuCoV-229E. Subcritical water extraction and resin adsorption techniques were applied to produce the extracts. The extracts were further characterised for their bioactive content, and three out of four extracts showed a high polyphenol content (>200 mg/g). The antiviral activity was assessed through viral infectivity and replication inhibition assays in human MRC-5 host cells. The results indicate that chicory leaf and red onion peel extracts demonstrated significant antiviral effects, with effective concentrations (EC_50_) of 61.43 µg/mL and 10.1 µg/mL, respectively. Olive pruning extract exhibited moderate activity, while vineyard pruning extract showed limited efficacy. These findings suggest that polyphenol-rich agricultural by-products could serve as sustainable sources for antiviral agents, warranting further investigation into their mechanisms of action and potential applications against other coronaviruses, including SARS-CoV-2.

## 1. Introduction

The agricultural sector is one of the biggest producers of residual biomass. For example, the olive oil and wine industry in Southern Europe wastes by-products estimated at 5000 and 3500 kton/year, respectively [1], and nearly half of the biomass constituting all the grown crops and vegetables is wasted, yielding approximately 400 million tonnes of dry matter of residues annually in Europe [2]. Thus, agricultural by-products represent an underexploited source of high-value bioactive compounds with significant potential for sustainable development. In recent years, the recovery and valorisation of these residues have attracted increasing attention, not only for their environmental benefits (by reducing waste and promoting a circular economy) but also for their potential applications in the food, nutraceutical and pharmaceutical industries [3,4]. Various sustainable extraction methods have been developed to efficiently recover these valuable compounds, and techniques such as subcritical water extraction (SWE), microwave-assisted extraction, hot-pressurised liquid extraction, supercritical fluid extraction, ultrasound-assisted extraction or enzyme-assisted extraction allow for the effective extraction of polyphenols and other bioactives while minimising the use of hazardous organic solvents and reducing energy consumption, adding economic value to agricultural residues [4,5,6,7]. In particular, SWE offers several advantages over other techniques, since it uses water under controlled temperature and pressure conditions, which eliminates the need for toxic organic solvents and significantly reduces the environmental impact. Moreover, by tuning the temperature and pressure, water’s polarity can be adjusted to optimise the solubility and selectivity for targeted bioactive compounds, resulting in high yields and improved extract purity while consuming less energy [7,8].

In parallel with the growing interest in sustainable valorisation, the rapid global outbreak of the severe acute respiratory syndrome coronavirus (SARS-CoV-2), commonly known as COVID-19, had a profound negative impact on the global population, intensifying the search for novel antiviral agents. SARS-CoV-2 is a member of a broader range of highly contagious human coronaviruses which are associated with respiratory symptoms ranging from the common cold (cough, fever) to pneumonia and bronchiolitis, spreading between people through close contact and via respiratory droplets produced from coughs or sneezes [9,10]. Human coronavirus 229E (HuCoV-229E) is one of the major viruses responsible for upper respiratory tract disorders, and is reported to be 65% identical to SARS-CoV-2 [9], thus being a suitable model for the initial screening of compounds with potential virucidal activity against SARS-CoV-2. HuCoV-229E can also cause severe respiratory disease in subsets of patients, with similar symptoms to SARS- and MERS-CoV [11].

Among the bioactive compounds present in agricultural by-products, polyphenols are known for their antiviral properties against a range of viruses. It has been postulated that hydroxyl and ester groups of polyphenols are required for antiviral activity so that the phenolics with five and more hydroxyl groups and 3, 4, 5-thrimethoxy derivatives show antiviral and anti-rabies activity. Additionally, alkyl-esters of gallic acid (gallates) and epicatechin contribute to anti-herpetic activity [12]. Some agricultural side streams, such as olive and vineyard prunings, chicory leaves and onion peels, are particularly rich in polyphenolic compounds with diverse bioactivities, including antiviral, antioxidant and anti-inflammatory properties, and represent a promising resource for developing high-value products [4,13,14]. However, the full potential of natural polyphenolic compounds from agricultural side streams remains underutilised due to the lack of sustainable, economically viable and effective technologies capable of preserving the polyphenols’ complexity and functionality while also delivering large quantities of active and safe products.

The aim of the present study was to evaluate and compare the antiviral activity of four polyphenol-rich extracts obtained by SWE and resin adsorption techniques from four agricultural by-products (red onion peels, vineyard prunings, olive prunings and chicory leaves) against HuCoV-229E as an initial screening to assess their potential efficacy against other viruses, including SARS-CoV-2, thereby assessing their potential as sustainable antiviral agents.

## 2. Results

### 2.1. Characterisation of Extracts

Four polyphenol extracts were produced using SWE for this study, namely from red onion peels, vineyard prunings, olive prunings and chicory leaves. Extraction yields were 23.00%, 28.58%, 20.90% and 41.18%, for red onion peels, vineyard prunings, olive prunings and chicory leaves, respectively. Chicory leaf and olive pruning extracts were further concentrated using adsorption resins; the final enriched fractions contained 10.45% and 40.11% of the initial dry weight of the deposited extracts for a final yield of 4.30% and 8.38%, respectively. Table 1 shows that extracts and concentrated extracts were rich in polyphenols, with three samples out of the four containing a very high polyphenol content (>200 mg GAE/g), and only vineyard prunings containing polyphenols at a moderate concentration level. The DPPH assay showed that the concentrated chicory leaf extract (PLX 386) exhibited the highest antioxidant activity among the tested samples, followed by the red onion peel extract (PLX 390), while the olive pruning (PLX 411) and the vineyard pruning (PLX 397) extracts presented more discrete antioxidant capacities.

Table 2 shows that all the four extracts contained a very rich profile of polyphenols, with specific components for each extract. Red onion peel extract (PLX 390) contained mainly quercetin and spireoside at high concentrations. Olive pruning extract (PLX-411) contained mainly oleuropein, tyrosol and verbascoside as the major polyphenols. Chicory leaf extract (PLX-386) was rich in chicoric acid, caftaric acid and caffeic acid, while vineyard pruning extract (PLX 397) predominantly contained resveratrol.

### 2.2. Inhibition of Viral Infectivity

**Figure 1:**
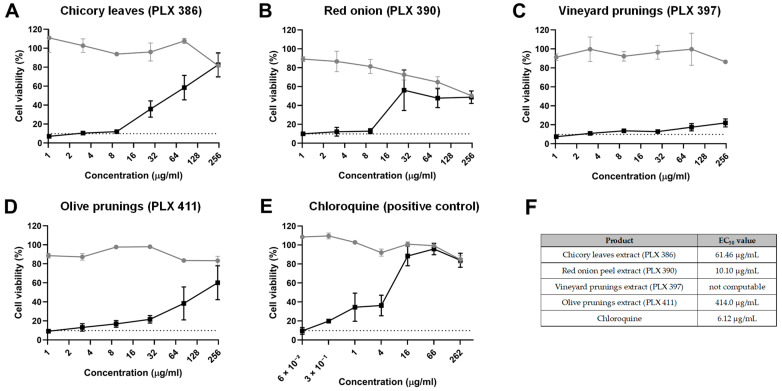
Inhibition of viral infectivity. Cell viability, as determined by an MTT assay, of MRC-5 cells treated with different concentrations of (**A**) chicory leaf extract (PLX 386), (**B**) red onion extract (PLX 390), (**C**) vineyard pruning extract (PLX 397), (**D**) olive pruning extract (PLX 411) and (**E**) chloroquine in the presence (black line and dots) or absence (grey line and dots) of a HuCoV-229E inoculum. The dashed line indicates the average viability of infected MRC-5 cells without any treatment. (**F**) Half maximal effective concentration (EC_50_) for each compound tested. Data are presented as the mean ± standard error mean (SEM).

To test the potential antiviral activity of the polyphenolic extracts against HuCoV-229E, we initially tested their ability to inhibit the virus CPE on host cells when co-treated with the virus, thus analysing the ability of these extract to inhibit the viral infectivity. This analysis of infectivity inhibition yielded interesting results with some of the polyphenolic extracts tested, displaying varying degrees of antiviral activity. Specifically, the chicory leaf extract (PLX 386) exhibited a reduction in CPE caused by HuCoV-229E at concentrations starting from 27.8 µg/mL, with an EC_50_ of 61.46 µg/mL. Notably, none of the concentrations tested caused a significant decrease in cell viability in cells treated with the extract alone (Figure 1A,F).

The red onion extract (PLX 390) showed improvement in the CPE caused by HuCoV-229E at concentrations starting from 27.8 µg/mL, although the effect was more pronounced, resulting in an EC_50_ of 10.1 µg/mL (Figure 1B,F). It is important to note, however, that this extract also showed significant cytotoxicity in MRC-5 cells at concentrations starting from 27.8 µg/mL. Therefore, the cell toxicity caused by the extract could potentially contribute to diminishing the replication capacity of the virus.

Regarding the vineyard pruning extract (PLX 397), it only showed a slight amelioration of the viral CPE at the highest concentrations tested. However, the EC_50_ could not be computed with the data obtained (Figure 1C,F).

As for the olive pruning extract (PLX 411), it also showed antiviral activity, albeit with a less pronounced curve and an EC_50_ of 414 µg/mL, with no significant cell toxicity exerted by the extract at the concentrations tested (Figure 1D,F).

The positive control, chloroquine, demonstrated a clear antiviral effect, with an EC_50_ of 6.12 µg/mL (Figure 1E,F).

### 2.3. Inhibition of Viral Replication

**Figure 2:**
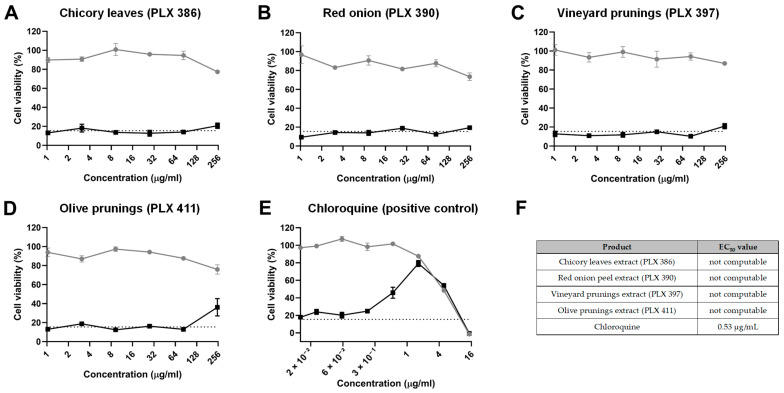
Inhibition of viral replication. Cell viability, as determined by an MTT assay, of MRC-5 cells treated with different concentrations of (**A**) chicory leaf extract (PLX 386), (**B**) red onion extract (PLX 390), (**C**) vineyard pruning extract (PLX 397), (**D**) olive pruning extract (PLX 411) and (**E**) chloroquine after being infected with a HuCoV-229E inoculum (black line and dots) or after being sham-infected (grey line and dots, CPE control). The dashed line indicates the average viability of infected MRC-5 cells without any treatment. (**F**) Half maximal effective concentration (EC_50_) for each compound tested. Data are presented as the mean ± standard error mean (SEM).

In order to discern whether the polyphenol extracts could also exert an antiviral effect once the host cells were already infected, we performed an experiment where cells were initially infected with HuCoV-229E for 4 h and then treated with the different extracts for 24 h. The results from this experiment did not show positive results for most of the extracts (Figure 2A,E). The only extract that showed a slight amelioration of the viral CPE was the olive pruning extract (PLX 397), at the highest concentration tested (250 µg/mL) (Figure 2A,E). However, with the data obtained, it was not possible to calculate an EC_50_ value.

As for the positive control (chloroquine), it was observed that a concentration of 1.63 µg/mL achieved maximum protection against viral CPE without causing cell toxicity, with an EC_50_ value of 0.53 µg/mL (Figure 2A,E), proving the sensitivity of the experimental procedure.

## 3. Discussion 

The present study demonstrated the infectivity inhibition potential of polyphenol-rich extracts derived from agricultural by-products against HuCoV-229E. Exceptionally high polyphenol levels (>200 mg EGA/g) were revealed in three out of the four extracts obtained, consistent with previous research indicating that agricultural by-products are rich sources of bioactive compounds, particularly polyphenols [4]. The high polyphenol content observed in the red onion peel, olive pruning and chicory leaf extracts suggests their potential for further investigation as functional ingredients in food, nutraceutical and pharmaceutical applications. Furthermore, the detailed polyphenol profiling demonstrated that each extract contained specific dominant compounds. Red onion peels were particularly rich in quercetin and spireoside, known for their strong antioxidant and antiviral properties [15]. Olive pruning extracts contained oleuropein, tyrosol and verbascoside, compounds associated with anti-inflammatory and cardiovascular health benefits [14]. Chicory leaves were found to be abundant in chicoric acid, while vineyard prunings predominantly contained resveratrol, a polyphenol with well-documented antiviral and cardioprotective properties [16].

The in vitro study on the antiviral capacity of the obtained polyphenolic extracts yielded interesting results. Specifically, EC_50_ values for inhibiting the infectivity of HuCoV-229E were obtained for the three extracts with higher TPC levels: chicory leaf extract, red onion extract and olive pruning extract. While the red onion extract (PLX 390) showed the lowest EC_50_ value, concentrations demonstrating antiviral activity also exhibited significant cytotoxicity, potentially complicating the interpretation of its antiviral efficacy. The chicory leaf extract (PLX 386), on the other hand, demonstrated promising antiviral effects and notably lacked significant cytotoxicity at concentrations displaying antiviral activity. In contrast, the vineyard pruning extract (PLX 397) displayed only a slight improvement in viral CPE, with insufficient data to compute an EC_50_. Similarly, the olive pruning extract (PLX 411) exhibited antiviral activity, albeit to a lesser extent compared with the chicory and red onion extracts, with an EC_50_ of 414 µg/mL.

These results are consistent with previous studies showing the antiviral properties of polyphenolic compounds, such as resveratrol [17], epigallocatechin-3-gallate [18], Bi121, a standardised polyphenolic-rich compound isolated from Pelargonium sidoides [19], quercitin [15], protocatechuic acid [20] and chicoric acid [16], thus reinforcing the antiviral potential of such compounds. Importantly, emerging evidence suggests that the antioxidant properties of polyphenols may play a critical role in reducing viral infectivity, since various viruses can cause an imbalance in the oxidative metabolism at the mitochondrial level, thus exacerbating viral pathogenesis by damaging the host’s cellular components, enhancing inflammatory responses and eventually causing cell death [21]. By neutralising reactive oxygen species, polyphenolic antioxidants may mitigate these effects and thereby inhibit the initial stages of viral entry [22]. In accordance with this, in our study, the samples with the lowest EC_50_ values were the ones with higher antioxidant activity.

Very few studies have explored the antiviral activity of extracts obtained via SWE. For instance, an SWE-derived extract from *Brassica juncea* showed antiviral activity against influenza virus A/H1N1 [23], and flucoidans extracted from *Nizamuddinia zanardinii* by SWE and other non-conventional methods exhibited strong antiviral activity against HSV-2 infection [24]. Notably, our study is the first to demonstrate that SWE-derived extracts from agricultural by-products exhibit antiviral activity against a human coronavirus, highlighting their potential as a novel source of bioactive compounds.

Previous studies investigating the antiviral activity of similar polyphenolic compounds and extracts have typically involved co-incubation of the virus with the test compounds. Importantly, the lack of an antiviral effect shown in our study when the extracts were administered 4 h after the viral infection suggests that the antiviral effect of the polyphenolic extracts appears to be limited to inhibiting viral infectivity.

In our study, the positive control, chloroquine, showed a clear antiviral effect, with a similar EC_50_ value for the inhibition of viral infectivity to that observed in other studies [19,25,26], and also showing antiviral potential when administered 4 h after the viral infection, thus suggesting that chloroquine is able to inhibit viral replication once the viral infection has occurred.

## 4. Materials and Methods

### 4.1. Plant Materials

Chicory leaves (fresh green leaves), *Cichorium intybus* var. *sativum*, were provided by farmers in the Bilzen area (Belgium) with the help of Sensus B.V. (Roosendaal, The Netherlands). The leaves were dried for two days in a drying oven at 40 °C. Red onion peels, *Allium cepa*, were collected in Castilla-la-Mancha (Spain) and were shipped to Celabor by Cartif Technology Centre (Valladolid, Spain). The peels were left in the field for 8 days to dehydrate prior to collecting and shipping to Celabor. Olive prunings (a mixture of leaves and branches) were collected by Saint André GAEC (Merschweiller, France) in the Provence–Alpes–Côte d’Azur region (France) from 25-year-old trees, *Olea europaea* var. *verdale aglandau*. The whole sample was air-dried and milled to a size of 0.5 cm to 10 cm. Vineyard prunings (branches) were collected by Chateau de la Martinette SCEA (Lorgues, France) in the Provence–Alpes–Côte d’Azur region (France) from 7-year-old trees, *Vitis vinifera* var. *caberbnet franc*. Those branches were also air-dried and milled to a size of 0.5 cm to 10 cm. Both olive and vine samples were shipped to Celabor by Chambre Régionale d’Agriculture Provence Alpes Côte d’Azur.

### 4.2. Extraction of Polyphenols by Subcritical Water Extraction

Polyphenol extracts were produced from the four feedstocks by subcritical water extraction (SWE) using a unit implemented at Celabor (Herve, Belgium). Dried raw materials were milled (2–4 mm sieve) and dropped into a 6 L stainless steel insert. The insert was introduced into a reactor, and the system was closed. Water was pumped and heated (120–150 °C) through a heat exchanger until the system was filled and reached the target pressure of 15 bars. Automatic valves were closed, and the recirculation pump was powered on to recirculate the water in the extraction loop at a flow rate of 1000 g/min. Recirculation was maintained for 30 min, then the liquid extract was cooled prior to system depressurisation. The total liquid extract was flushed in the collector using a nitrogen flow to drain the system. The final extracts were recovered from the extract collector. Two complete cycles of extraction were performed and pooled together prior to the drying step (spray-drying) to obtain the polyphenol extracts. The yield of extraction was determined as follows: the weight of the total dry extract was divided by the amount of dry feedstock multiplied by 100.

### 4.3. Enrichment in Polyphenols Using Adsorption Resin Chromatography

Two polyphenol liquid extracts (chicory leaves and olive prunings) were directly subjected to a further enrichment step using hydrophobic adsorption resin to increase their purity in batch mode. After some optimisation trials, Amberlite XAD7 was selected for enrichment in polyphenols in the chicory leaf extracts whereas Amberlite XAD16 was used for the olive pruning extracts. Specifically, liquid extracts were poured on activated resins in a 25 L reactor at a 1/10 (resin–liquid) ratio and left overnight under mechanical stirring for adsorption. Then, the resin loaded with polyphenols was recovered by filtration on a 200 mesh filter cloth and washed with fresh water. Polyphenols were then desorbed from the resin with 96% ethanol under mechanical stirring for 2 h followed by filtration, evaporation under a vacuum, and spray-drying to obtain the enriched polyphenol extracts. The mass yield of the concentration process was determined as follows: the weight of the total dry enriched extract was divided by the total amount of dry raw extract multiplied by 100.

### 4.4. Characterisation of Polyphenol Extracts

#### 4.4.1. Total Phenolic Content (TPC) Determined by the Folin–Ciocalteu Method

The total phenolic content (TPC) was determined according to the Folin–Ciocalteu method [27] with gallic acid as the reference compound. For this, 25 mg of the extract was dissolved in 5 mL of an ethanol/water mixture (50:50) and agitated for 15 min using ultrasound to facilitate solubilisation. The obtained solutions were diluted according to the expected quantity. In total, 100 µL of the diluted solution was added to 1.5 mL of distilled water, 400 µL of Folin–Ciocalteu reagent, 600 µL of Na_2_CO_3_ at 200 g/L and 2.4 mL of distilled water. The reaction mix was homogenised and left in the dark at room temperature for 2 h. The absorbance was then measured at 760 nm using a UV–visible Genesys 150 spectrophotometer (Thermo Fisher Scientific Inc., Waltham, MA, USA) against a calibration curve of gallic acid ranging from 25 to 400 ppm. The results are expressed as mg of gallic acid equivalent (GAE) per gram of sample (mg GAE/g).

#### 4.4.2. Determination of the Antioxidant Activity by the DPPH Method

The 2,2-diphenyl-1-picrylhydrazyl (DPPH) assay is based on electron transfer. DPPH is a stable free radical which has a strong purple colour in solution. In the presence of compounds capable of either transferring an electron or donating hydrogen, DPPH becomes pale yellow. The disappearance of purple colour can be measured spectrophotometrically and is related to the antioxidant capacity of the sample tested.

The assay was performed with extracts dissolved in methanol. For this, 5 mg of DPPH was dissolved in 25 mL of methanol. A calibration curve was made for different gallic acid solutions from 1 to 10 mg/L. Gallic acid is a natural molecule present in a wide variety of plants; this compound is a reference in antioxidant power determination which has the capacity to trap free radicals. For this, 150 µL of each point of the calibration curve and 150 µL of 8 dilutions from the sample were distributed on a microplate. Then 75 µL of the DPPH solution was added, and measurement of the absorbance at 515 nm was performed on a spectrophotometer (Tecan infinite 200 series; Tecan Group Ltd., Männedorf, Switzerland) after 30 min of incubation away from light at room temperature. The results were obtained by considering the IC_50_ of the samples compared with gallic acid and are given as mg GAE per gram of dry extract.

#### 4.4.3. Quantitation of Major Polyphenols by UPLC-MS-MS

The identification and quantitation of polyphenols in the dried extracts were performed by ultra-high-performance liquid chromatography coupled with mass spectrometry, using an Acquity UPLC Xevo-TQ system (Waters Corporation, Milford, MA, USA) system equipped with a Acquity BEH Shield RP18 column. The gradient profile of the mobile phase (ammonium formate buffer (Solvent A) and acetonitrile (Solvent B)) was 0–7.20 min, 95–80% A; 7.20–10 min, 80–75%; 10–11.5 min, 75–50% A; 11.5–12 min, 50–0% A; 12–12.5 min, 0% A (isocratic); 12.5–14 min, 0–95% A; 14–16.5 min, 95% A (isocratic), with a constant flow of 0.5 mL/min. The temperature of the column was fixed at 40 °C and the vials containing the samples were maintained at 15 °C during the analysis. For this, 3.5 µL of each sample was injected, and the analyses were monitored by MassLynx Software version 4.2 (Waters, Milford, MA, USA) Polyphenols in the samples were identified by their *m*/*z* values and fragment patterns after comparing them with an internal database containing 100+ standards. After the identification of the compounds of each extract, they were quantified by calibration curves made with external standards of the chosen molecules with concentrations between 0.25 and 10 mg/L. The results are expressed in mg/g.

### 4.5. Cell Culture and Viral Strain

The MRC-5 cell line (lung-derived fibroblasts) was obtained from American Type Culture Collection (ATCC; CCL-171) Manassas, VA, USA. Cells were maintained at 37 °C and 5% CO_2_ in a humidified atmosphere in EMEM (ATCC; 30-2003) supplemented with 10% FBS (Sigma; F7524, St. Louis, MO, USA) and antibiotics (Corning; 30-002-CI, Corning, NY, USA), and were trypsinised every 3–4 days before reaching 90% confluence. Human coronavirus HuCoV-229E was obtained from ATCC (VR-740) and amplified in MRC-5 cells according to the ATTC’s specifications.

### 4.6. Viral Infectivity Inhibition Experiments

MRC-5 cells were seeded in 96-well plates at a density of 5000 cells/well 48 h before the treatments.

For each extract, a 50 mg/mL stock solution was prepared in DMSO (Sigma; D4540) and filtered (0.22 µm). The stock solution was diluted to 500 µg/mL in PBS (Sigma; D5773), and serial dilutions (1/3) of the 500 µg/mL solution were prepared in PBS + 1% DMSO.

In total, 250 µL of each dilution was mixed with 250 µL of HuCoV-229E diluted in a viral medium (EMEM + 2% FBS and antibiotics), with a final HuCoV-229E concentration in the mixes of 1000 TCID_50_/mL. As a negative control, PBS + 1% DMSO was mixed with HuCoV-229E. The test substance–virus mixes were incubated at 25 °C for 30 min. Then, the cell medium was aspirated, and the cells were inoculated with 100 µL of the test substance–viral mixes. Cells were incubated with the mixes at 35 °C and 5% CO_2_. After 4 h, the cell medium was aspirated and replaced with the viral medium without the virus, and the cells were further incubated at 35 °C and 5% CO_2_. Five days after inoculation, the MTT assay was performed. Chloroquine (Sigma; C6628) was used as a positive control, using the same procedure described above for the extracts.

In order to determine the cytotoxicity of each treatment concentration without the virus, cytotoxicity controls were performed for all the concentrations of the extracts and chloroquine, mixing 250 µL of each dilution with 250 µL of the viral medium without the virus, and treating the cells with 100 µL of these mixes for 4 h. For all treatments, the final concentration of DMSO in the medium during the 4 h of inoculation was 0.5%, and the experiments were performed at least in triplicate.

### 4.7. Viral Replication Inhibition Experiments

MRC-5 cells were seeded in 96-well plate at a density of 5000 cells/well 48 h before the treatments.

The cell medium was aspirated, and cells were inoculated with 100 µL of HuCoV-229E diluted in a viral medium with a final HuCoV-229E concentration of 1000 TCID_50_/mL. Cells were incubated with the virus at 35 °C and 5% CO_2_. After 4 h, the inoculums were aspirated, and serial dilutions of the extracts diluted in the viral medium with 0.5% DMSO were added to the wells. Cells were incubated with the extracts at 35 °C and 5% CO_2_ for 24 h. Then, the cell medium was aspirated and replaced with the viral medium, and the cells were further incubated at 35 °C and 5% CO_2_. Five days after inoculation, the MTT assay was performed. Chloroquine was used as a positive control, using the same procedure described above for the extracts.

In order to determine the cytotoxicity of each treatment concentration without the virus, cytotoxicity controls were performed for all the concentrations of the extracts and chloroquine, treating MRC-5 cells with 100 µL of the viral medium without the virus for 4 h before incubating them with different concentrations of the extracts and chloroquine. For all treatments, the final concentration of DMSO in the medium during the 4 h of inoculation was 0.5%, and the experiment were performed at least in triplicate.

### 4.8. Determination of Cell Viability by the MTT Assay

Cell viability was quantified by the MTT assay, a colorimetric assay that measures the activity of enzymes that transform the MTT molecule to insoluble formazan, which has a purple colour [28]. After treatments, the cell medium was aspirated, and 100 µL of MTT 1 mg/mL (Sigma; M2128), diluted in EMEM, was added to the wells. The cells were then incubated at 35 °C for 2 h. The MTT solution was aspirated, formazan crystals were dissolved in 100 µL of DMSO, and absorbance was measured at 570 nm in a SPECTROstar Nano absorbance microplate reader (BMG Labtech, Ortenberg, Germany). The mean values of non-infected controls were considered as 100% cell viability.

### 4.9. Data Calculations

Half maximal effective concentration (EC_50_) values, defined as the compound concentration that inhibits the cytopathic effect (CPE) caused by the viral infection by 50%, were obtained by non-linear regressions, using GraphPad Prism software (v. 10.2.1). All graphs were generated using GraphPad Prism software (v. 10.2.1).

## 5. Conclusions

In summary, our work highlights the potential of agricultural by-product-derived polyphenol extracts as antiviral agents and demonstrate that sustainable extraction approaches, such as SWE, can effectively obtain these bioactive compounds while minimising environmental impact and supporting the circular economy by valorising agricultural residues. Further research on the most promising extracts should focus on determining their mechanism of antiviral action, assessing the reduction in viral infectious titres and their efficacy in other viral strains such as SARS-CoV-2 and non-enveloped viruses, and validating their efficacy and safety profiles in animal models.

## Figures and Tables

**Table 1:** Total phenolic content and antioxidant power of each extract, measured by the Folin–Ciocalteu and DPPH assays.

Sample	Feedstock	TPC ± SD (mg GAE/g)	DPPH ± SD (mg GAE/g)
**PLX 390**	Red onion peels	202.36 ± 3.10	62.58 ± 2.72
**PLX 397**	Vineyard prunings	82.02 ± 0.62	32.01 ± 4.55
**PLX 386**	Chicory leaves	210.35 ± 2.83	102.59 ± 6.12
**PLX 411**	Olive prunings	206.55 ± 4.77	38.695 ± 3.54

**Table 2:** Identification and concentration of phenolic compounds in each extract.

**PLX 390—Red Onion Peels**		**PLX 397—Vineyard Prunings**	
**Metabolite**	**Concentration (mg/g)**	**Metabolite**	**Concentration (mg/g)**
Protocatechuic acid	12.55 ± 1.05	Protocatechuic acid	0.86 ± 0.01
Quercetin	42.70 ± 4.01	Gallic acid	0.89 ± 0.01
Isorhamnetin	0.43 ± 0.05	Resveratrol	3.77 ± 0.44
Tamarixetin	0.83 ± 0.04	Piceatannol	0.17 ± 0.01
Quercetine-7-O-glucoside	0.80 ± 0.02	Epicatechin	0.25 ± 0.03
Isoquercitrin	0.05 ± 0.01	Catechin	0.52 ± 0.06
Spireoside	12.78 ± 1.78	Polydatin	0.11 ± 0.01
**PLX 386—Chicory Leaves**		**PLX 411—Olive Prunings**	
**Metabolite**	**Concentration (mg/g)**	**Metabolite**	**Concentration (mg/g)**
Chicoric acid	30.97 ± 1.74	Oleuropein	63.50 ± 7.55
Caftaric acid	10.66 ± 0.16	Verbascoside	21.94 ± 0.89
Caffeic acid	8.63 ± 0.51	Tyrosol	40.72 ± 3.08
Luteolin-7-O-glucuronide	2.98 ± 0.77	Cynaroside	2.65 ± 0.60
Luteolin	0.44 ± 0.19	Oleacein	1.53 ± 0.23
Chlorogenic acid	2.46 ± 0.35		

## Data Availability

The raw data supporting the conclusions of this article will be made available by the authors on request.

## Abbreviations

The following abbreviations are used in this manuscript:

ATCC	American Type Culture Collection
COVID-19	Coronavirus disease 2019
CPE	Cytopathic effect
DMSO	Dimethyl sulfoxide
DPPH	2,2-Diphenyl-1-picrylhydrazyl
GAE	Gallic acid equivalent
EC_50_	Half maximal effective concentration
EMEM	Eagle’s minimum essential medium
FBS	Fetal bovine serum
MERS-CoV	Middle East respiratory syndrome coronavirus
MTT	3-(4,5-Dimethylthiazol-2-yl)-2,5-diphenyltetrazolium bromide
PBS	Phosphate-buffered saline
SARS-CoV-2	Severe acute respiratory syndrome coronavirus 2
SEM	Standard error of the mean
SWE	Subcritical water extraction
TCID_50_	Tissue culture infectious dose 50%
TPC	Total phenolic content
UPLC-MS-MS	Ultra-high performance liquid chromatography coupled with mass spectrometry
